# The Role of Cortisol and Dehydroepiandrosterone in Obesity, Pain, and Aging

**DOI:** 10.3390/diseases13020042

**Published:** 2025-02-01

**Authors:** Nikolina Erceg, Miodrag Micic, Eli Forouzan, Nebojsa Nick Knezevic

**Affiliations:** 1 Department of Anesthesiology, Advocate Illinois Masonic Medical Center, Chicago, IL 60657, USA; nerceg01@gmail.com (N.E.); micic.usmle@gmail.com (M.M.);; 2 Faculty of Medicine, University of Belgrade, 11000 Belgrade, Serbia; 3 Department of Anesthesiology, University of Illinois, Chicago, IL 60612, USA; 4 Department of Surgery, University of Illinois, Chicago, IL 60612, USA

**Keywords:** cortisol, DHEA, DHEAS, cortisol-to-DHEA ratio, obesity, aging, acute pain, chronic pain

## Abstract

Obesity, chronic pain, and aging are prevalent global challenges with profound implications for health and well-being. Central to these processes are adrenal hormones, particularly cortisol and dehydroepiandrosterone (DHEA), along with its sulfated form (DHEAS). Cortisol, essential for stress adaptation, can have adverse effects on pain perception and aging when dysregulated, while DHEA/S possess properties that may mitigate these effects. This review explores the roles of cortisol and DHEA/S in the contexts of obesity, acute and chronic pain, aging, and age-related diseases. We examine the hormonal balance, specifically the cortisol-to-DHEA ratio (CDR), as a key marker of stress system functionality and its impact on pain sensitivity, neurodegeneration, and physical decline. Elevated CDR and decreased DHEA/S levels are associated with worsened outcomes, including increased frailty, immune dysfunction, and the progression of age-related conditions such as osteoporosis and Alzheimer’s disease. This review synthesizes the current literature to highlight the complex interplay between these hormones and their broader implications for health. It aims to provide insights into potential future therapies to improve pain management and promote healthy weight and aging. By investigating these mechanisms, this work contributes to a deeper understanding of the physiological intersections between pain, aging, and the endocrine system.

## 1. Introduction

Obesity, pain, and aging represent significant healthcare challenges globally. Over the past three decades, obesity rates have increased significantly, more than doubling between 1990 and 2022. In 2022, one in eight people worldwide were living with obesity, amounting to a total of over one billion individuals affected [1]. Pain is the most common symptom for seeking medical consultation. It is estimated that 1 in 5 adults worldwide experience pain, and 1 in 10 are diagnosed with chronic pain each year [2]. It is proposed that chronic pain affects an estimated 20% of adults globally [3]. Simultaneously, the population is growing rapidly, with projections indicating that by 2030, 1 in 6 people globally will be aged 60 or older [4]. Given the growing number of individuals impacted by these conditions, extensive research has been dedicated to understanding their physiological and clinical implications.

While acute cortisol release aids short-term adaptation, its prolonged elevation may contribute to adverse effects, including weight gain, altered pain perception, and accelerated aging. In contrast, the roles of adrenal androgens DHEA/S, known for their anti-glucocorticoid and potential anti-aging effects, remain poorly explored in these contexts [5].

In this narrative review, we aim to provide a comprehensive overview of the role of cortisol and DHEA/S in obesity, pain, aging, and age-related diseases, analyzing the current literature to elucidate how these hormones influence these processes. Ultimately, this work seeks to provide insights that can inform clinical practice and guide future research toward more effective approaches to obesity, pain management, and aging, improving the well-being of individuals living with obesity, pain, or age-related morbidities. Furthermore, we will examine current therapeutic strategies and explore potential pathways for future interventions aimed at mitigating age-related changes in the human body.

## 2. Regulation of Cortisol Secretion and Its Effects

**Figure 1:**
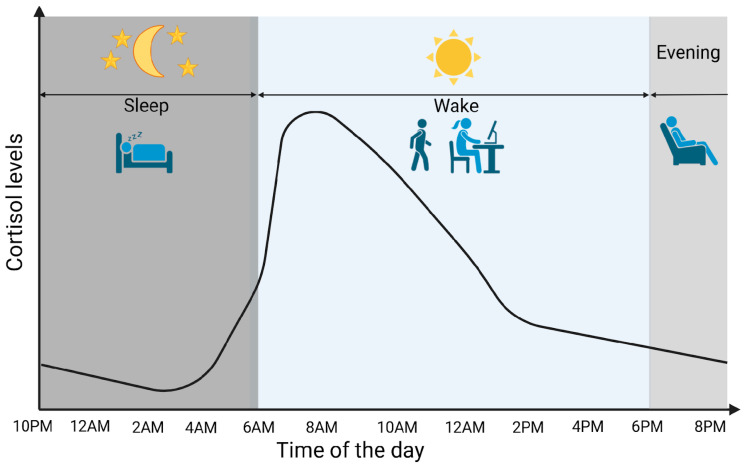
Cortisol circadian secretion pattern. Created in BioRender. Erceg, N.; Micic, M. (2024), https://BioRender.com/d14h073 (accessed on 10 December 2024).

**Figure 2:**
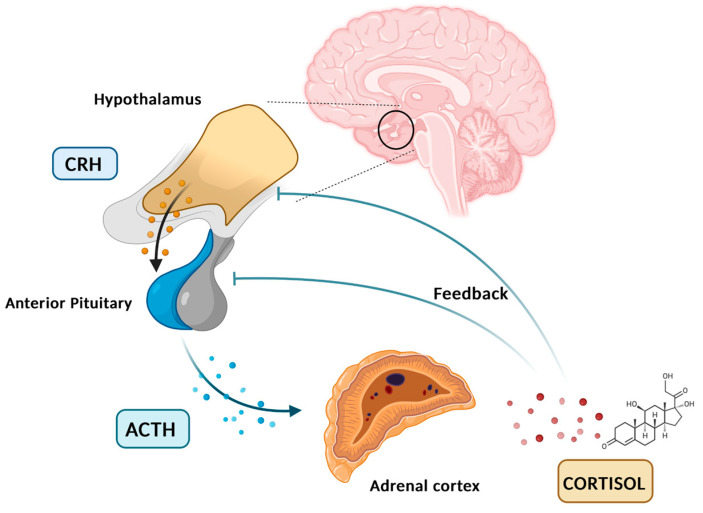
HPA axis, cortisol feedback loop. Created in BioRender. Erceg, N.; Micic, M. (2024), https://BioRender.com/p52o659 (accessed on 10 December 2024).

Cortisol is a glucocorticoid (GC) produced by adrenal glands, mainly by the zona fasciculata. It plays a vital role in various processes, including regulating stress response and maintaining homeostasis. Cortisol synthesis and secretion are controlled by the HPA axis, a key system in the body’s response to stress [5]. In the absence of stressors and under normal conditions, cortisol is secreted in circadian and ultradian rhythms, controlled by the suprachiasmatic nucleus located in the hypothalamus. Figure 1 graphically represents the diurnal cortisol pattern [6]. When a stressor is detected or when there is a psychological response, the HPA axis is activated. Corticotropin-releasing hormone (CRH) is secreted by the paraventricular nucleus of the hypothalamus into the hypophyseal portal system. This stimulates the anterior pituitary gland to secrete adrenocorticotropic hormone (ACTH). ACTH then circulates through the bloodstream to the adrenal cortex, where it attaches to melanocortin receptors, leading to the synthesis and release of cortisol [7]. Cortisol synthesis and secretion are regulated by a negative feedback loop mechanism (Figure 2) [8].

In the circulation, cortisol molecules exist in a free, active form (5%) and bound inactive form (95%). GCs secreted into the bloodstream bind to the cortisol-binding globulins (CBGs) or albumin. In order to exert their effect, free soluble cortisol molecules must bind to specific cytosolic, glucocorticoid, and mineralocorticoid receptors (GRs and MRs). Upon ligand binding to those cytosolic receptors, ligand–receptor complexes are translocated to the nucleus where they act as transcriptional factors influencing gene expression [9].

Cortisol plays a vital role in mediating stress response in humans. Moreover, cortisol is involved in the regulation of metabolism, inflammatory responses, and immune function. In order to increase glucose concentration and improve its availability in the blood, cortisol promotes gluconeogenesis and glycogenolysis, while also inducing insulin resistance in peripheral tissues. Cortisol has anti-inflammatory and immunosuppressive properties [9]. It also influences the cardiovascular system by maintaining blood pressure [6]. Although cortisol has a critical effect in managing the stress response, prolonged exposure to elevated cortisol levels seen in chronic stress conditions or during the aging process could have detrimental effects on health such as the increased risk of developing various chronic diseases, pain sensitivity, obesity, immune suppression, depression, and cognitive impairments [5]. Prolonged or excessive elevation of cortisol levels can trigger adaptive mechanisms in the body, leading to a decrease in the sensitivity or number of GRs. This phenomenon, known as GR down-regulation or resistance, impairs the normal binding of cortisol to these receptors. As a consequence of recurrent stress exposure, the body’s cortisol regulation system may eventually become compromised [10]. The dysregulation of cortisol signaling can lead to various pathological conditions, highlighting the importance of maintaining hormonal balance [11].

## 3. Role and Secretion Regulation of DHEA/S

DHEA and its sulfated form, DHEAS, are steroid androgenic hormones produced by the adrenal glands, predominantly by the zona reticularis and significantly less so by the zona fasciculata [5]. While DHEAS is exclusively produced by adrenals, DHEA can originate from gonads and other extra-adrenal regions as well [12]. ACTH, intra-adrenal, and extra-adrenal factors influence DHEA/S secretion [13]. DHEA secretion shows diurnal variations with the lowest levels observed in the morning, while DHEAS levels tend to remain stable during the day mostly due to a lower clearance rate. DHEA/S are bonded to albumin in the circulation [12].

**Figure 3:**
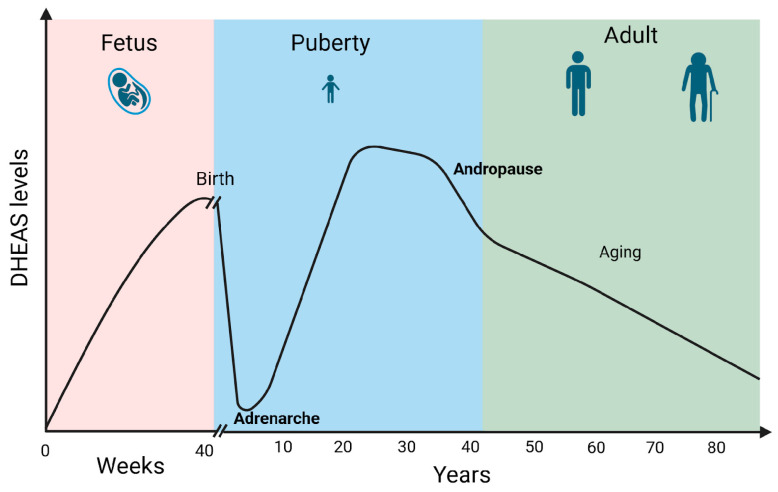
DHEAS levels during life. Created in BioRender. Erceg, N.; Micic, M. (2024), https://BioRender.com/l50y500 (accessed on 10 December 2024).

DHEA/S secretion has been shown to be sex- and age-dependent. Males show higher DHEAS levels compared to females. Furthermore, DHEA levels typically decline with age. Fetal and early postnatal life is accompanied by high levels of DHEA/S. Soon after birth, the DHEA/S level falls quickly, remaining low until adrenarche between 10 and 12 years of age. The peak of DHEA/S levels is reached during the 30 s. Androgen levels then gradually drop by 1–2% annually. By the fifth decade, both male and female androgen levels had already decreased by 60% of the peak production in a process called andropause. Androgen levels then continue to drop throughout life reaching 20–30% of the peak production in the eighth and ninth decade of life [14] (Figure 3). Moreover, DHEA levels seem to decrease in certain stress-related conditions which can impact overall well-being and pain perception [5].

**Figure 4:**
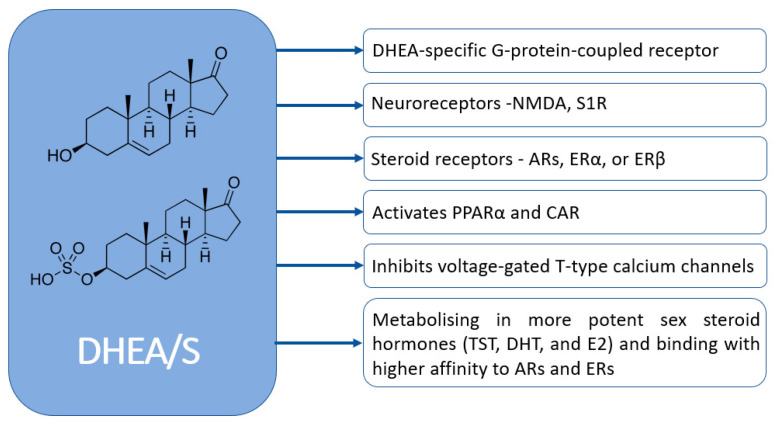
Mechanisms of DHEA/S actions (NMDA—N-methyl-d-aspartate; S1R—sigma-1 receptor AR—androgen receptor; ER—estrogen receptors; TST—testosterone; DHT—dihydrotestosterone; E2—estradiol; PPARα—peroxisome proliferator-activated receptor; CAR—constitutive androstane receptor).

**Figure 5:**
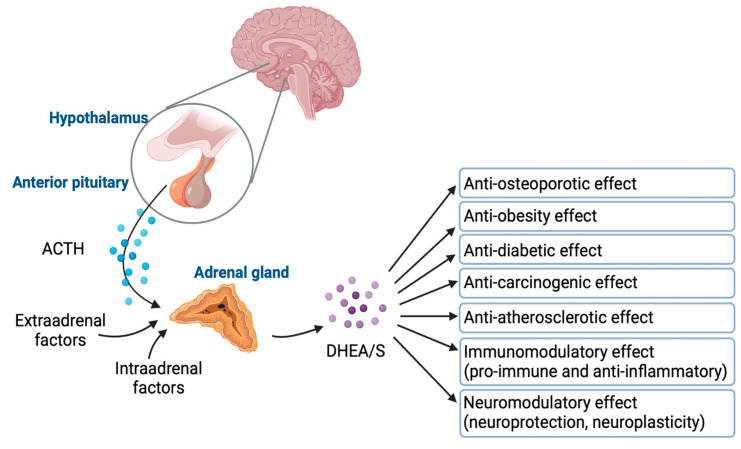
DHEA/S regulation and their effects. Created in BioRender. Erceg, N.; Micic, M. (2024), https://BioRender.com/m89f416 (accessed on 10 December 2024).

DHEA/S have pleiotropic effects. Despite their significance, the full extent of their physiological effects and the mechanisms through which they operate are not completely understood. Figure 4 graphically represents how DHEA/S may exert their physiological effects [15]. DHEA/S are mostly considered as a precursor hormone due to their weak androgenic effects. After being transformed into a potent androgen or estrogen, DHEA/S exert a full biological effect. Also, DHEA/S function as neurosteroids, highlighting their versatility. Even though DHEA/S are known for their anti-aging properties, they perform a variety of roles within the body, including providing neuroprotection, promoting neurite outgrowth, exerting antagonistic effects on oxidative agents and glucocorticoids, and modulating the immune system as well as lipid and glucose metabolism [14,16]. DHEA/S exert anti-obesity, anti-diabetic, anti-carcinogenic, anti-atherosclerotic, anti-inflammatory, anti-osteoporotic, and pro-immune effects as well (Figure 5) [12]. This wide range of actions underscores their potential impact on maintaining physiological balance and responding to stressors [16]. Lower levels of DHEA/S are associated with age-related diseases, obesity, frailty, cardiovascular events, and mortality, while higher DHEA/S levels correlate with improved well-being, health outcomes, psychological status, functional abilities, and longevity [17]. Moreover, there is growing evidence of the DHEAS role in pain modulation that will be discussed later.

## 4. Cortisol–DHEA/S Balance

The cortisol-to-DHEA ratio (CDR) has been proposed to precisely reflect HPA axis functionality [18]. Currently, DHEA and its sulfated counterpart, DHEAS, are recognized for their important roles in counteracting elevated cortisol levels and in the biosynthesis of androgens and estrogens [16]. With their anti-glucocorticoid properties acting both centrally and peripherally while counteracting the negative effects of cortisol, they have been involved in neuroprotection, immunomodulation, and metabolism regulation [5].

**Figure 6:**
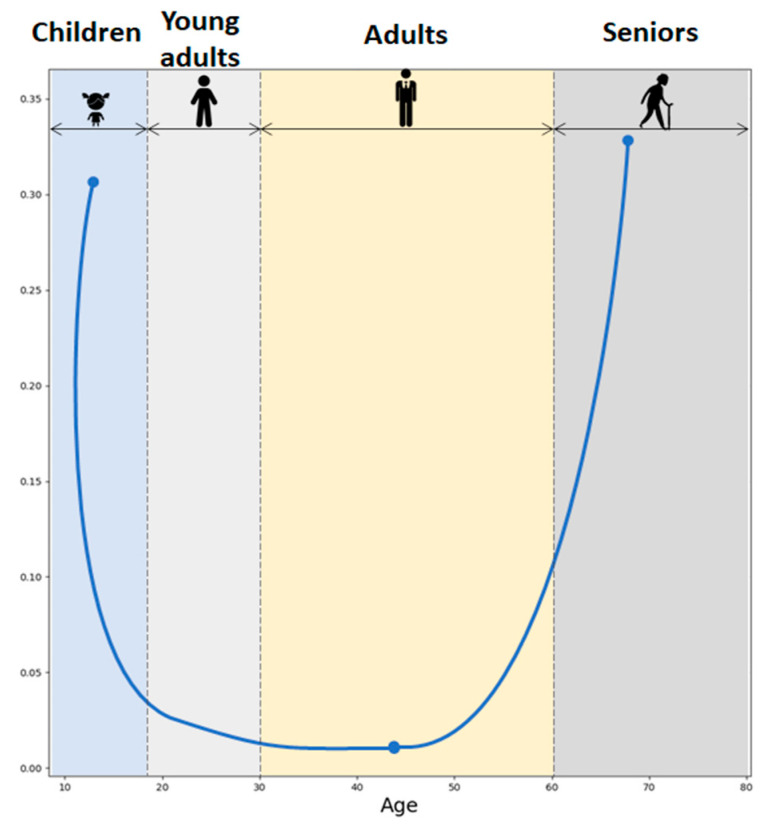
Cortisol-to-DHEAS molar ratio across the lifespan.

Due to cortisol and DHEA diurnal variation, the most pronounced CDR elevation has been observed in the morning. Throughout the lifespan, the CDR exhibits a U-shaped curve as illustrated in Figure 6, based on the values reported in the study by Yen and colleagues [19]. Moreover, studies have shown that higher molar CDR has been observed during both physiological and pathological aging [20]. Higher CDR has also been associated with enhanced neurotoxicity, with implications for the development of age-related diseases in the aged population [17]. Even though GCs are crucial in managing the body’s fight-or-flight response, exposure to elevated levels of GCs could exert negative effects on human health. GCs have the effect of dampening many immune system activities. In contrast, DHEAS predominantly boosts immune function and exhibits properties that counteract the effects of GCs. The relationship between cortisol, DHEAS, and immune function is well documented. High cortisol levels and a high CDR are associated with reduced natural killer cell activity (NKA), a critical component of the immune response. Conversely, higher DHEAS levels are linked to improved NKA, particularly in premenopausal women. This suggests that maintaining a balance between cortisol and DHEAS is essential for immune health, which can influence pain perception and management [21].

Moreover, it is thought that DHEAS may mitigate some of the physiological impacts associated with cortisol, particularly those related to stress [22]. CDR has gained attention as a valuable marker for understanding the balance between catabolic and anabolic processes, as well as for evaluating chronic stress and overall health [23]. Chronic stress and chronic diseases impact the HPA axis, causing changes in how cortisol and DHEA respond. Research has shown that individuals with higher lifetime stress exposure tend to exhibit reduced cortisol responses but increased DHEA responses when faced with acute stress. Therefore, altered HPA axis functioning and CDR may contribute to increased susceptibility to stress-related disorders, chronic pain, and age-related diseases, highlighting the importance of understanding the cumulative impact of stress on hormonal balance [5].

## 5. Obesity

Clinically, obesity is diagnosed in individuals whose Body Mass Index (BMI) exceeds 30 kg/m². It is characterized by an excessive expansion of adipose tissue through both hyperplasia and hypertrophy of adipocytes in order to store excessive amounts of lipids. As adipose tissue enlarges, it secretes adipokines and pro-inflammatory cytokines in a dysfunctional manner, coupled with an increased release of free fatty acids that contributes to chronic low-grade inflammation and the onset of insulin resistance, dyslipidemia, and other obesity-related metabolic disorders. Obesity also involves a disrupted HPA axis, altered cortisol secretion profiles, and diurnal salivary cortisol rhythms [24]. GCs participate in several processes within adipose tissue, including adipogenesis, metabolism, inflammation, and adipokine production. Prolonged, excessive cortisol levels have been associated with higher body weight, obesity, the expansion of central (particularly visceral) adipose tissue, a reduction in subcutaneous adipose tissue, and impairment of brown adipose tissue [25,26]. Notably, the expansion of visceral adiposity has been more strongly linked to obesity-related metabolic disorders such as insulin resistance, dyslipidemia, diabetes mellitus, and cardiovascular complications. Chronic corticosteroid use directly correlates with abdominal weight gain, and obesity itself is linked to a significantly higher use of glucocorticoids [24,27].

Although blood, saliva, and urine cortisol levels have not shown a consistent relationship with obesity—possibly due to the circadian pattern of cortisol secretion—hair cortisol concentration (HCC) has demonstrated a stable association. Specifically, HCC correlates positively with BMI and the waist–hip ratio, with an observed 9.8% increment in HCC linked to a 2.5 kg/m^2^ higher BMI [27]. As hair grows at approximately 1 cm per month, HCC reflects long-term cortisol exposure and thereby indicates HPA axis function over an extended period. Higher HCC values have been found in obese individuals compared to those who are overweight or of normal weight, and these elevated HCC measurements are also associated with an increased prevalence of metabolic syndrome [24]. Even though heightened GC levels drive the aforementioned changes in the human body, responses to identical GC increments can vary among individuals because of differences in GC sensitivity. Consequently, GC responses depend not only on the concentration of GCs but also on their availability and on the sensitivity of the receptors [27].

While it is not yet fully elucidated, both the GR and the MR appear to be significantly involved in the pathogenesis of obesity [28]. Polymorphisms in the NR3C1 gene, which codes for GR, lead to variations in GC sensitivity, metabolic profiles, and body composition, thereby representing potential risk factors for obesity-related diseases and adverse cardiometabolic profiles. Two polymorphisms, N363S and BclI, are associated with increased GC sensitivity and correlate with unfavorable lipid profiles, abdominal obesity, hyperinsulinemia, and hypertension. Conversely, the ER22/23EK and 9β polymorphisms show greater resistance to GC and are linked to more favorable metabolic profiles, including less central obesity. In men, these polymorphisms have been associated with increased height and muscle strength, while in women, they are linked to smaller waist and hip circumferences [29]. GC sensitivity can be assessed in vivo through the dexamethasone suppression test or in vitro using reverse transcription–quantitative polymerase chain reaction (RT-qPCR) [27]. These tests aid in evaluating the risk factors for various metabolic diseases and in predicting responses to GR-targeted therapies, as well as the likelihood of adverse effects—such as disturbances in glucose homeostasis—during treatment with adrenal cortex hormones [30]. However, further research is necessary to confirm their clinical utility.

MR also plays a fundamental role in adipose tissue, particularly during adipocyte differentiation, in regulating adipokine secretion and autophagy, and in corticosteroid-induced adipogenesis [31]. As adipocytes mature, MR expression increases. MR activation has detrimental effects on adipose tissue, including adipocyte hypertrophy, increased macrophage infiltration and pro-inflammatory polarization, upregulated expression of pro-inflammatory adipokines, mitochondrial dysfunction, excessive reactive oxygen species (ROS) production, and impaired browning processes [32]. When MR activation is excessive—such as in obesity, where adipocyte MR expression is elevated—it promotes the dysfunctional processes characteristic of metabolic syndrome, including increased fat mass, endothelial dysfunction, oxidative stress, and inflammation [32,33]. On the other hand, studies show that MR knockdown in primary human visceral preadipocytes disrupts their differentiation and reduces PPARγ expression, a key transcriptional regulator of adipogenesis. Obese patients exhibit increased MR expression in adipocytes, both in visceral and subcutaneous adipose tissue with higher expression among visceral adipocytes. Research indicates that dexamethasone application elevates leptin and adiponectin levels while reducing pro-inflammatory cytokines (e.g., IL-6, TNF, and MCP1). Conversely, aldosterone heightens the expression of pro-inflammatory cytokines (PAI-1 and MCP1) and suppresses adiponectin expression [28]. Several studies following treatment with MR antagonists have shown significant reductions in central fat mass and BMI, restoration of insulin sensitivity, lowered fasting triglyceride levels, and improved brown adipose tissue function. Thus, MR antagonists exert anti-adipogenic effects by inhibiting adipose differentiation and reducing PPARγ expression [32].

Elevated DHEA levels have been associated with a reduced prevalence of obesity in both men and women, as well as with less abdominal fat accumulation in men. In contrast, low DHEA levels correlate with increased adiposity and a higher incidence of various age-related diseases, including obesity, type 2 diabetes, and atherosclerosis [34]. Multiple studies examining the impact of DHEA on adipose tissue suggest that it inhibits adipocyte proliferation and differentiation, stimulates triacylglycerol hydrolysis, enhances glucose uptake, suppresses 11-βHSD1 activity and leptin synthesis, and upregulates adiponectin gene expression [35].

Obesity is a significant risk factor for numerous health conditions and diseases. It contributes to cardiovascular diseases (hypertension, coronary arterial disease, heart failure, and stroke) and metabolic disorders (type 2 diabetes mellitus, dyslipidemia, and metabolic syndrome) and it is closely linked to respiratory disorders (obstructive sleep apnea, obesity hypoventilation syndrome, and asthma). It increases the risk of developing various cancers including breast, colorectal, endometrial, pancreatic, esophageal, prostate, and kidney cancer. Furthermore, excess weight significantly impacts the musculoskeletal system, increasing the risk of osteoarthritis, gout, and chronic back pain. Gastrointestinal and liver diseases (non-alcoholic fatty liver disease and gallbladder disease) are also more prevalent in individuals with obesity. Obesity significantly affects reproductive health and hormonal balance, increasing the risk for polycystic ovary syndrome, infertility, and pregnancy complications. Moreover, psychological conditions such as depression and anxiety and impaired cognition have been associated with obesity. Chronic low-grade inflammation caused by obesity also weakens the immune system, increasing susceptibility to infections and severe outcomes from illnesses [36].

Obesity and pain are intricately connected, with longitudinal studies identifying obesity as a significant risk factor for the development of chronic pain. Research indicates that individuals with obesity exhibit a lower pain threshold to electrical and mechanical stimuli, along with a higher prevalence of pain, greater pain extent, and more persistent pain complaints in both adults and adolescents. As BMI increases, reports of pain, particularly low back pain, become more common. Furthermore, obesity is strongly associated with the onset, progression, and severity of osteoarthritis, often necessitating surgical intervention. Notably, evidence suggests that weight loss can significantly alleviate chronic pain in individuals with obesity, underscoring the importance of weight reduction in pain relief strategies [37].

## 6. Pain

Pain serves as a warning of tissue damage, signaled by specific receptors and fiber systems that extend from the periphery to the brain [38]. Acute pain, typically lasting from a few minutes to less than six months, is triggered by injury, surgery, illness, trauma, or other medical procedures and is usually resolved once the underlying cause is treated or healed. It aims to produce behavior that will prevent further injury [39]. While acute pain can be beneficial to the affected patient by signaling injury or harm, chronic pain does not always serve this purpose, and it may be present despite the initial cause being resolved. Chronic pain can result from various causes, including injury, inflammation, underlying diseases, or idiopathic conditions, and it may have elements of central sensitization [39,40]. Timely management of acute pain lowers the risk of developing chronic pain syndromes [39]. It is believed that cognitive and emotional factors can play a crucial role in pain perception due to the interconnected brain regions involved in pain processing, attention, expectation, and emotional regulation [39]. Although the underlying pathophysiology of different pain conditions remains unclear, recent studies have increasingly focused on gaining a deeper understanding of the physiological processes involved in pain, with a particular emphasis on neurobiological mechanisms [41].

Recent studies have shown the role of GR expression in human pain-sensing dorsal root ganglion (DRG) neurons. Deep single-soma RNA sequencing and RNAscope have revealed robust expression of GRs in DRG neurons, with levels significantly exceeding those observed in traditional stress-responsive brain regions like the hippocampus [42]. These findings underscore the unique role of DRG neurons in stress and glucocorticoid signaling. Mechanistically, GR activation in sensory neurons has been shown to drive hyperexcitability and aberrant plasticity. Specifically, glucocorticoid receptor-dependent transcriptional programs promote neurite sprouting and peripheral axon regeneration, which may contribute to nociceptive hypersensitivity and pain persistence [43]. This aligns with clinical observations correlating cortisol with pain sensitivity, providing essential molecular insight by linking these changes to specific transcriptional mechanisms in sensory neurons.

Chronic pain affects over 50 million Americans, but treatments remain inadequate due to a limited understanding of its underlying biological mechanisms [3]. Over time, chronic pain significantly impacts quality of life by restricting mobility, disrupting sleep, causing depression and anxiety, and reducing overall productivity [40]. In recent years, chronic musculoskeletal pain conditions have gained growing medical interest. Pain biomarkers could help identify altered biological pathways and phenotypic expressions, offer insights into treatment targets, and detect at-risk patients for early intervention [3]. Continuous research has demonstrated that cortisol abnormalities correlate with pain, whether acute (e.g., postoperative) or chronic [44]. The next section will provide a detailed explanation of the connection between acute and chronic pain and its relationship with cortisol and DHEA/S.

### 6.1. Cortisol, DHEA, and Acute Pain

The link between cortisol and stress has been studied, with acute pain recognized as a form of stress. Research has demonstrated that cortisol abnormalities correlate with pain, whether acute (e.g., postoperative) or chronic [44]. Salivary cortisol levels have been proven to be effective in reflecting pain responses across a range of procedures, from abdominal surgery to molar extractions, underscoring its value as a marker for stress- and pain-related research. In one study of 102 subjects, salivary cortisol jumped from a median of 7 ng/mL to 17 ng/mL after tooth extraction [45]. Bariatric surgeries utilizing robotic, minimally invasive techniques have also been shown to significantly reduce physiological stress compared to traditional open surgeries. In one study, patients undergoing these procedures experienced lower self-reported pain levels, which corresponded with reduced cortisol levels (mean of 114 ng/mL vs. 76.3 ng/mL 24 h postop) [46]. This pattern has been consistently observed in the studies reviewed. A study of 60 patients found that a novel bupivacaine protocol during cardiac surgery not only improved subjective pain outcomes as measured by a visual analog scale (3.4 ± 1.1 vs. 1.1 ± 0.6) but also reduced ICU stays with these improvements quantitatively linked to lower levels of free cortisol (631 ± 505 µg vs. 401 ± 297 µg at 24 h) [47]. Indeed, this novel, preemptive intervention seems to have improved pain and cortisol levels as much as minimally invasive surgery. However, evidence suggests that cortisol levels themselves may influence acute pain sensitivity.

Abnormal cortisol levels appear to influence pain perception in surgical patients, with notable effects observed even in response to acute cortisol changes. Preoperative stress-induced cortisol fluctuations have demonstrated a particularly significant impact. For example, a laboratory study found that mice with an increased adrenal gland index (~160 mg/kg vs. ~125 mg/kg), induced by preoperative stressors (such as food deprivation, restraint stress, tilted cage, cold swimming, and overnight illumination) experienced prolonged pain recovery following surgery (indicated by earlier paw withdrawal threshold to mechanical stimuli and intolerance to cold stimuli) [48]. In addition, studies have shown that acutely abnormal cortisol enhances pain sensitivity in general but not necessarily when induced by surgery. A randomized, double-blind, and placebo-controlled study with 100 subjects found that 20 mg oral hydrocortisone decreased female subjects’ visceral pain threshold as measured by rectal pressure under time (37.3 mmHg before treatment vs. 31.8 mmHg after treatment); however, this effect was less pronounced in men (34.2 mmHg before treatment vs. 32.8 mmHg after treatment) [49]. Another study of 176 subjects also found that after baseline cortisol was controlled for, increased cortisol suppression after DEX administration and lower recovery cortisol levels were associated with higher pain ratings during a cold pressor test (Bs = −2.42 to −17.82; with Bs = change in the pain rating for each 1-unit increase in cortisol levels) as well as with reduced conditioned pain modulation via heat stimulus (Bs = −0.92 to −1.68) [50]. This phenomenon is believed to result from dysfunction of the HPA axis, which can arise from various physiological or psychological factors [51].

Interestingly, a study of 201 patients found that blood cortisol concentrations measured on the first postoperative day serve as a prognostic marker for the risk of developing persistent postoperative pain syndrome, with this effect being more pronounced in older individuals (B = 0.86 ± 0.08, odds ratio 2.36) [52]. Advancing age and increasing frailty have been associated with a gradual decline in hypothalamic sensitivity, resulting in elevated cortisol levels and a disrupted diurnal rhythm. These changes weaken the negative feedback mechanisms of the HPA axis [53]. In contrast, a review of 32 and 21 studies examining the predictive factors for postoperative pain intensity and analgesic use, respectively, revealed that age frequently exhibited a negative correlation with both analgesic consumption (as noted in six studies) and postoperative pain intensity (also in six studies). Only one study indicated a positive correlation between age and postoperative pain [54].

Growing evidence suggests that emotional distress caused by impending surgery may affect cortisol levels. A recent study of 71 patients undergoing total joint arthroplasty found that the pre-surgical and 24 h post-surgical samples showed a marked increase in cortisol levels (>200 ng/mL) when compared to non-surgical controls [55]. The researchers postulated that it is possible that psychological stress experienced during the pre-surgical period is sufficient to elevate patients’ serum cortisol levels [40]. Another study demonstrated that pain hypervigilance served as a strong predictor of subjective acute postoperative pain (17% explained variance) in patients undergoing chest malformation correction surgery [56]. This explained variance increased to 35% when combining the hypervigilance parameters plus heat pain threshold, cortisol suppression, and somatoform symptoms [56]. Additionally, a prospective study of 168 patients found that pain score at discharge had a significant negative correlation with baseline cortisol levels (r = −0.142) and heightened psychological stress (measured by DASS stress total)—often associated with minority status and single individuals—contributed to the development of increased pain in patients with traumatic injuries [57].

A new area of interest is the connection between DHEA, cortisol, and pain perception. In terms of acute pain, a study of 20 female anorexia nervosa patients found that thermal pain threshold correlated negatively with DHEA (r = −0.53) and positively with cortisol/DHEA (r = 0.76) and cortisol/DHEAS (r = 0.54) [58]. These findings were corroborated by another study involving 20 patients undergoing total knee arthroplasties. Postoperative pain scores exhibited a negative correlation with DHEA, as demonstrated by a Spearman rho of −0.64 for 24 h pain scores at rest and −0.63 for 48 h pain scores at rest [59]. Additionally, postoperative pain scores were positively correlated with the CDR, showing a Spearman rho of 0.60 for 24 h pain scores at rest and 0.60 for 48 h pain scores at rest [59]. These results emphasize the role of an increased cortisol-to-DHEA balance in enhancing pain perception during the postoperative period [59].

### 6.2. Cortisol, DHEA, and Chronic Pain

Dysregulation of the stress response system can contribute to the persistence and exacerbation of chronic pain [10]. Research has demonstrated that prolonged stress exposure can result in an attenuated cortisol awakening response (CAR), which serves as a predictor for pain intensity and fatigue severity observed in various chronic pain syndromes, including fibromyalgia, chronic fatigue syndrome, and persistent pelvic pain. This neuroendocrine dysregulation is frequently linked to enhanced nociception and increased inflammatory processes, contributing to the pathophysiology of chronic pain conditions [60,61]. For example, individuals experiencing chronic musculoskeletal pain across multiple anatomical areas frequently show reduced cortisol levels upon waking, along with a less pronounced diurnal decline. These patterns suggest an underactive HPA axis, reflecting a potential disruption in the body’s stress response system [62]. In fibromyalgia, numerous investigations have identified irregularities in the HPA axis function. Research by Riva and colleagues demonstrated that fibromyalgia patients exhibited reduced cortisol concentrations in the morning and a diminished CAR when compared to controls. These alterations in cortisol dynamics may contribute to pain sensitization and other symptoms [63]. Moreover, GRc, as an essential regulator within the HPA axis, mediates cortisol’s anti-inflammatory and immunosuppressive effects. Dysregulated GR signaling is also linked to chronic pain conditions, such as fibromyalgia and neuropathic pain, where altered receptor expression or sensitivity impairs the regulation of inflammation and nociceptive processing, contributing to pain persistence [51].

DHEA and its sulfated form, DHEAS, have not been extensively explored as biomarkers for chronic pain [64]. DHEAS has been identified as an endogenous modulator of Kv7 channels, exhibiting a unique mechanism of action that diminishes M-current suppression. This newly discovered form of Kv7 channel modulation decreases the sensitivity of M-currents to stimulation by Gq-coupled receptors. Such a modulatory effect holds significant potential for attenuating heightened neuronal activity, positioning DHEAS as a promising candidate for therapeutic interventions [65].

Several observational studies have identified links between DHEA levels and chronic pain disorders. In a comprehensive study utilizing data from the Midlife in the United States (MIDUS) cohort, Li et al. analyzed blood levels of DHEA/S, in relation to chronic pain among 1216 adults aged 34 to 84 years. They found that women with chronic pain had significantly lower DHEAS levels compared to pain-free women, with a dose–response relationship observed for pain interference. Interestingly, no association was found between DHEA/S and chronic pain in men. Emerging evidence indicates that sex hormones, particularly estrogen and androgen, significantly influence chronic pain conditions, with testosterone linked to lower pain sensitivity and estrogen exhibiting complex associations with pain. Low levels of estradiol and progesterone in women may heighten pain vulnerability by impairing the endogenous opioid system, while decreased DHEAS levels could predict chronic pain due to their role in sex hormone production [41].

Comparable results indicating decreased DHEA/S levels in chronic pain conditions have been observed in studies focusing on specific disorders. Freitas et al. observed lower salivary DHEA levels in women with fibromyalgia compared to healthy controls. For fibromyalgia syndrome (FMS), patients exhibited reduced cortisol concentrations (10.10 ± 4.08 μg/dL) compared to the control group (11.78 ± 3.6 μg/dL). Similarly, serum DHEAS levels were notably lower in FMS patients (89.93 ± 53.96 μg/dL) than in controls (143.15 ± 107.92 μg/dL). These findings suggest alterations in the HPA axis and steroid hormone production in individuals with fibromyalgia [66]. In a pilot study led by Grimby-Ekman involving individuals with chronic neck pain, plasma DHEAS levels were generally lower in the pain group compared to the control group. The baseline levels of age and sex-adjusted estimated DHEAS were 2.3 µmol/L (95% confidence interval [CI] 1.34 to 4.08) for the pain group, while the control group had levels of 3.7 µmol/L (95% CI 1.90 to 7.14) Additionally, the response of DHEAS to exercise differed between the groups. In the control group, there was a tendency for DHEAS levels to decrease following exercise, whereas in the chronic pain group, DHEAS levels either slightly increased or remained unchanged [67]. Also, experimental studies provide further evidence for DHEA’s role in pain modulation. Kibaly et al. demonstrated that DHEA administration reduced mechanical hypersensitivity in a mouse model of neuropathic pain. The analgesic effects were mediated through sigma-1 receptors and associated with decreased spinal neuroinflammation [68].

Understanding the roles of cortisol and DHEAS in chronic stress and chronic pain has significant clinical implications. Therefore, assessing cortisol and DHEAS levels could help identify individuals at risk of developing chronic pain and those who may benefit from stress management interventions. Further empirical studies are essential to explore the potential of hormone replacement therapy targeting DHEA/S pathways as a novel approach to chronic pain management [69].

## 7. Changes in Cortisol and DHEA/S Secretion/Levels Related to Aging

### 7.1. Aging Process

Aging is defined as a progressive loss of physiological integrity leading to functional impairment and an increased likelihood of death [70]. It involves a gradual decline in an organism’s ability to adapt to its environment accompanied by a diminished capacity for tissue and organ regeneration across the body [71]. Aging is caused by several molecular mechanisms such as DNA damage, mutations and epigenetic alterations, accumulation of damaged and dysfunctional proteins, changes in cell metabolism, greater levels of oxidative stress and mitochondrial dysfunction, and finally, cellular senescence. All of these mechanisms play a crucial role in aging, making affected cells more prone to various stressors [72].

The hallmarks of the aging of the endocrine system are disruption of negative feedback loops and alteration in hormone production with a decrease in hormone levels being the most common [73]. It is accompanied by numerous changes involving a gradual decline in organ functions such as cognition, reduction in bone density, lower muscle mass, increased insulin resistance, immune dysfunction, and many others [17]. Even though the aforementioned changes are physiological, the presence of inflammation, chronic diseases, and low nutrition leads to an increased risk of developing age-associated diseases [73]. It is important to assess risk factors and causes of age-associated diseases in order to prevent and treat them efficiently.

### 7.2. HPA Axis, Cortisol, and Aging Process

Extensive quantitative and qualitative changes in the HPA axis as well as its regulation and hormone secretion patterns have been observed throughout the process of aging. As a result of adrenal cells’ senescence, multiple structural and functional changes are seen in aging adrenal glands [74] (Table 1). When aged adrenal glands were analyzed, adrenal atrophy with reduced zona reticularis, concomitantly increased zona fasciculata, and discontinuous zona glomerulosa were shown. All of these changes imply altered hormone production which will be discussed further in the next section. Additionally, an increased incidence of adrenal nodules/tumors has been observed in the aged population when compared to younger individuals [75].

Aging is accompanied by higher basal HPA axis activity, HPA axis disruption, increased mean cortisol concentration levels, normal secretion patterns with a flattened diurnal secretion slope, decreased diurnal cortisol variations, attenuated morning awakening response, earlier morning level peak, and higher late-afternoon, early-evening, and night nadir of cortisol with the shortening of the evening cortisol quiescent period [17,76,77,78,79] (Table 2). The HPA negative feedback loop disruption seen is characterized by decreased responsiveness to ACTH, inadequate drops of hormones after exogenously applied hydrocortisone or dexamethasone during suppression tests, and decreased number and sensitivity of GRs [17,20,76,80,81]. Boscaro et al. investigated ACTH responses after hydrocortisone administration in younger (18–26 years old) and aged (65–99 years old) men. Besides both groups having similar cortisol responses, the group of aged men showed an insignificant decline in ACTH in the first 15 min after the hydrocortisone administration, followed by a marked decline from 15 to 60 min after the administration. On the contrary, the group of younger subjects showed a more prominent decline in ACTH levels within the first 15 min after the hydrocortisone administration and a less pronounced decline from 15 to 60 min [80]. The study conducted by Carroll and colleagues revealed that older participants were more likely to not have adequate drops of cortisol after dexamethasone administration compared to the younger ones [81]. Besides HPA axis disruption, a possible explanation for Carroll’s results could be an increase in the number of cortisol-producing adenomas that are more commonly seen in the aged population. Incongruous results have been observed in studies that investigated CRH sensitivity in older adults, with some showing that exogenously applied CRH exhibited no age-related difference, while others proved an increased sensitivity to it [74]. Interestingly, the activity of an enzyme that converts inactive cortisone to active cortisol, 11β-HSD1, has been shown to increase with age in the central nervous system, bones, skeletal muscles, and skin [17,82]. Therefore, adverse effects of high cortisol levels present in the peripheral tissues could be seen in the elderly [17]. Decreased steroid sulfation and thus inactivation, an increased concentration of GC-precursor 11-deoxy cortisol, and lower cortisol-binding globulins, especially in males, may be additional factors contributing to higher cortisol levels in the aging population [83,84,85]. Age-related changes in cortisol profiles and the HPA axis may lead to an increased risk of developing age-related diseases such as osteoporosis, neurocognitive deficits, Alzheimer’s disease, insulin resistance, and many others [17,78].

### 7.3. DHEA/S and Aging

DHEA/S levels show a notable decline with advancing age, particularly in the elderly. Individuals in their 80s or 90s remain with just 20–30% of their DHEA peak secretion [14]. Studies have shown that advancing age is associated with decreased activity of 17,20-lyase, an enzyme responsible for converting 17α-hydroxy pregnenolone to DHEA. Moreover, the decrease in IGF-I and IGF-II have been proposed to affect adrenal androgen levels [19]. Interestingly, it has been shown that levels of 11-deoxy androgen, the precursor of DHEA, remain stable during aging [86,87]. The exact mechanism underlying lower DHEA and DHEAS and stable 11-deoxy androgen is not yet fully known but the key reason for this discrepancy might be the reduction in zona reticularis [74]. Besides low plasma concentrations of DHEA observed in the elderly, Henley et al. measured cortisol and DHEA in the saliva of subjects. They found that older participants had significantly higher cortisol AUC, reduced overall DHEA levels (with the most prominent reduction in the morning), and lower DHEA AUC. Moreover, with increasing age, the diurnal DHEA slope became less steep and the CDR linearly increased. The higher CDR seen in the morning might be a significant factor contributing to the aging process [88].

### 7.4. Factors Altering Hormone Secretion in the Elderly and the Implications

Aberrant hormone production and secretory-associated senescence phenotype in the aging adrenals affect the body’s functions and metabolism, inducing the senescent phenotype of the aging individual. The altered hormone patterns increase oxidative stress and genomic damage resulting in telomere shortening and stress-induced cellular senescence of neighboring and distant cells [74].

Higher cortisol levels have been associated with age-associated diseases, while DHEA exerts a positive effect with higher levels associated with better survival in males [89]. The natural decline in DHEA/S levels over the lifespan has prompted researchers to investigate the potential consequences of this age-related reduction. As these pro-hormones diminish, there is growing evidence to suggest that their depletion may contribute to the deterioration of metabolic and physical functions commonly associated with aging. This deficiency is hypothesized to play a role in the onset and progression of various age-related disorders, potentially through mechanisms involving immune system dysregulation, chronic inflammation, and increased oxidative stress [90,91]. Additionally, chronic stress accelerates aging and promotes the development of age-related diseases. Individuals with accelerated aging had typical impairment of cortisol circadian rhythm with consistently high levels of cortisol throughout the day [92]. Researchers have found that social disadvantages are associated with increased activity of the HPA axis [93] and those who are experiencing social stress and daily discrimination have been identified as having morning hypocortisolism and flatter diurnal cortisol slopes [94]. Higher diurnal cortisol profiles among people with low social support and poor resilience are associated with an increased risk of developing chronic diseases [95]. Also, adverse childhood experiences are linked to HPA axis dysregulation and the presence of chronic diseases in older age [96]. Therefore, healthy aging could be affected by several factors including all kinds of chronic stress (social stress, emotional stress, etc.), lifestyles, genetics, chronic diseases, and environments. The next section will discuss several age-associated diseases and evaluate hormone levels in relation to the clinical data.

## 8. Cortisol and DHEA/S Connections with Age-Related Diseases

### 8.1. Dementia

Aging can exert a harmful effect on the human brain which could be clinically presented as a broad spectrum of symptoms, from mild cognitive impairment to dementia. Dementia is defined by progressive or persistent loss of cognitive functioning which interferes with a person’s daily life and activities [97]. Currently, Alzheimer’s disease (AD) is the most common form of dementia, affecting 10% of individuals over 65 and 50% of those over 85. Alzheimer’s disease is characterized by the loss of neurons, synapse loss, and the accumulation of amyloid plaques and neurofibrillary tangles. Risk factors for developing AD are age, positive family history, presence of the APOE ε4 allele, genetic variations, chronic diseases, and lifestyle [98]. Although the exact neuropathological mechanism of AD is still being studied, researchers have been paying a lot of attention to the connection between chronic stress and AD, recognizing chronic stress as an important risk factor for AD. It has been noted that those who experienced a higher degree of perceived stress had a significantly higher risk for AD development [99].

**Figure 7:**
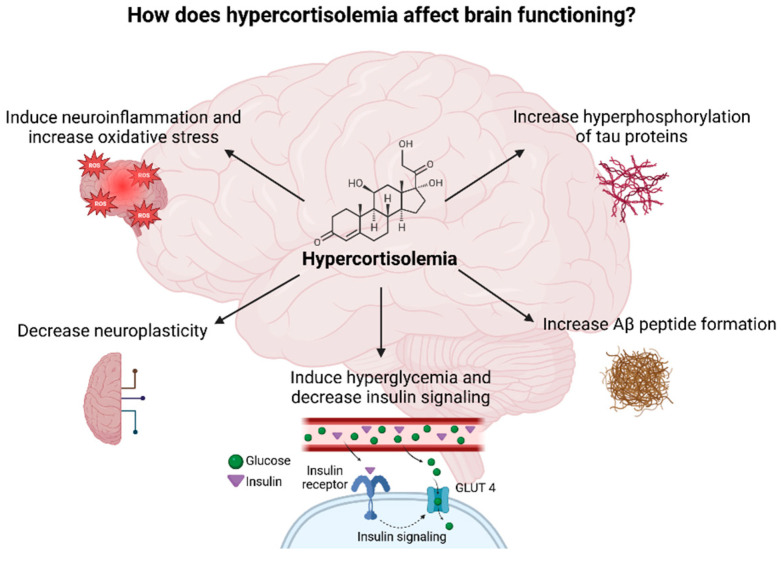
Diagram illustrating the potential impacts of elevated cortisol levels on brain function Created in BioRender. Erceg, N.; Micic, M. (2024), https://BioRender.com/w11y943 (accessed on 10 December 2024).

Chronic stress occurs when the stressor persists over an extended period of time and negatively affects the physiological and psychological functioning of the individual. Chronic stress accelerates aging and promotes the development of age-related diseases, especially AD. Exposure to chronic stressors affects the HPA axis in a similar way as aging does due to high cortisol levels seen in both cases [6,100,101]. Hypercorticolism observed during chronic stress affects brain function through several mechanisms (Figure 7). Moreover, high cortisol levels lead to a decrease in the volume of the HC, PFC, and temporal–parietal–occipital regions and to an increase in the volume of the amygdala which clinically presents as anxiety and cognitive impairment [17,102,103]. Importantly, the HC and PFC regulate cortisol’s diurnal rhythm, predominantly inhibiting the limbic–HPA axis activity, whereas the amygdala seems to activate the stress response [17].

The etiopathogenetic mechanism of worse cognition with increasing levels of cortisol is explained by the theory of two types of receptors present in the brain that could bind GC—GR and MR. While GRs are expressed ubiquitously across the neurons in the central nervous system, MR expression is more restricted on several structures including the hippocampus (HC), prefrontal cortex (PFC), paraventricular nucleus, nucleus of the solitary tract, locus coeruleus, and amygdala. Also, MRs are expressed in glial cells. MRs have a higher affinity to the cortisol molecule compared to the GRs, resulting in the occupation and activation of the MRs at the basal GC levels. GRs become occupied and activated when GC levels reach a certain level during a circadian peak or stress. The HC neurons have both types of receptors, and based on their activation, different cellular responses are generated. Therefore, sustained activation of GRs in the HC neurons seems to negatively affect synaptic plasticity and reduce brain-derived neuronal factor (BDNF) levels, leading to impaired memory performance. Contrarily, the activation of MRs is required for HC plasticity [17,104]. Lupien et al. conducted longitudinal research on healthy-aged individuals (60–90 years old), dividing them into three groups based on their basal cortisol levels and cortisol slopes—Increasing/High, Increasing/Moderate, and Decreasing/Moderate cortisol groups. First, they found that individuals from the Increasing/High cortisol group had significantly diminished declarative memory performance compared to the other two groups. Next, they performed an MRI on individuals from the Increasing/High and Decreasing/Moderate cortisol groups, finding that the Increasing/High cortisol group had a 14% lower volume of HC compared to the other group. Therefore, showing that the degree of cortisol elevation over time and current basal cortisol levels correlated with the individuals’ HC volume suggests that higher cortisol levels are associated with the reduction in HC volume and with learning and memory impairments [105]. Moreover, higher CSF cortisol levels and CSF CDR were associated with higher p-tau and tau CSF levels and lower amygdala and insula volumes at the baseline [106].

Studies have shown that cortisol could exhibit a neurotoxic effect on neurons by stimulating their death and inducing a reduction in dendritic length and degeneration through increased vulnerability to metabolic and vascular injuries [107]. Using near-infrared spectroscopy, researchers have demonstrated that higher hair cortisol concentrations are negatively associated with frontal lobe oxygenation [108]. Moreover, FDG PET analysis of participants who had normal cognition, mild cognitive impairment, or AD showed that higher plasma cortisol levels were associated with lower glucose metabolism in medial and lateral parietal regions in all three groups [102].

In contrast, DHEA stimulates neuronal long-term potentiation, neurite growth, and survival by protecting neurons from structural and functional damage. It also promotes glial survival [109]. Therefore, an increased CDR could have a detrimental effect on the human brain which subsequently results in the development of age-related neurodegenerative diseases.

Studies have shown that higher salivary cortisol, nighttime cortisol, hair cortisol concentration, diurnal and nocturnal urinary cortisol, and CSF cortisol are associated with worse cognitive functions in aged adults. Contrastingly, larger diurnal drops and higher CARs were associated with better cognitive performance [103,106,108,110,111]. Ennis et al. investigated whether cortisol dysregulation is related to the risk of developing AD. They took samples of creatinine (Cr) and 24 h urinary free cortisol (UFC) from the volunteers participating in the Baltimore Longitudinal Study of Aging and calculated the UFC/Cr levels and UFC/Cr variability. The results showed that participants who had increased UFC/Cr levels and increased UFC/Cr variability predicted an increased risk of AD on average 6 years before the disease onset [112]. Next, Ouanes et al. demonstrated that higher CSF cortisol levels predicted a more marked cognitive decline over 36 months, while higher cortisol and DHEAS predicted more pronounced disease progression over the same period [106].

Comparing the patients with AD and healthy subjects (HSs), researchers found that patients with AD had significantly higher cortisol levels (in plasma, saliva, urine, and CSF) than HSs and it correlated negatively with memory performance [103,104,106,113,114]. Also, studies reported that patients with AD are more likely to have lower DHEA levels both in CSF and plasma [106]. More research needs to be carried out to determine the role and effect of cortisol and DHEA on the development of AD. Even though it is still unclear whether cortisol is the cause or just correlative of AD, it could be assessed as a potential biomarker of the increased risk of AD or even as a target of preventive and/or therapeutical strategies in the future.

### 8.2. Depression

Aging is associated with several conditions that increase the risk of depression, including chronic medical illnesses, cognitive impairment, sleep disturbances, circadian rhythm disruptions, reduced social support, social isolation and loneliness, limited physical activity, and impaired overall functioning. These factors collectively contribute to chronic stress and diminished functional capacity [115,116,117]. Both aging and chronic stress, as well as depressive disorders, are characterized by dysregulation of the HPA axis, typically manifested by elevated basal cortisol levels and a flatter diurnal cortisol slope. Studies indicate that higher cortisol levels and decreased HPA axis sensitivity correlate with poorer cognitive outcomes, vascular and degenerative dementias, depression, and anxiety, while a flattened diurnal cortisol slope has been linked to adverse emotional and physical health outcomes [17,118,119]. Moreover, age- and stress-related changes in brain structure and function are associated with increased vulnerability to depression, cognitive decline, and anxiety [17].

Depression itself is marked by disrupted pulsatile cortisol secretion, hypercortisolemia, a reduced CAR, elevated urinary free-cortisol levels, increased cerebrospinal fluid CRH, and lower DHEA/S levels. Additionally, patients with depression show higher cortisol responses after dexamethasone administration and an enhanced cortisol response during post-stressor recovery [17,120,121,122]. An elevated CDR may further predict delays in recovery [123]. Alterations in cortisol and DHEA/S levels have also been observed in older adults with depression. Belvederi et al. reported that older adults with depression had significantly higher basal cortisol levels throughout the day—especially during the evening and night—compared to healthy, age-matched controls. The authors suggested that this pattern may be due to an age-associated reduction in MR function, which, in younger patients, compensates for impaired GR activity commonly seen in depression. As this compensatory mechanism wanes with age, HPA axis overactivity is likely exacerbated [124]. Elevated cortisol levels in older adults with depression have also been linked to significantly smaller hippocampal volumes compared with non-depressed controls [19]. Furthermore, older depressed individuals may exhibit decreased dexamethasone availability for suppressing plasma cortisol levels as well as elevated basal cortisol and heightened stress-related cortisol secretion [125]. Notably, significant differences in DHEAS levels and CDR have been observed between depressive and non-depressive centenarians. A further decrease in DHEAS levels among depressed individuals reinforces the additional role of depression in the age-related increase in CDR [126].

Aging is associated with higher consumption of various medications, many of which—such as antihypertensives, antiparkinsonian agents, chemotherapy drugs, hormones, and benzodiazepines—have been implicated in the onset or exacerbation of depressive symptoms [127]. Among these, GCs are particularly notable due to their widespread therapeutic use and potential to induce depression as a side effect. This condition, known as glucocorticoid-induced depression, arises from the exogenous administration of GCs, which mimic the physiological and pathological effects of endogenous hypercortisolemia [128]. GCs are among the most commonly prescribed medications, valued for their potent anti-inflammatory and immunosuppressive properties. They are used to manage a wide range of inflammatory and autoimmune disorders, including rheumatoid arthritis, asthma, inflammatory bowel disease, and sarcoidosis. Additionally, they are utilized in treating adrenal insufficiency, preventing transplant rejection, alleviating cerebral edema, and addressing other conditions requiring anti-inflammatory or hormone replacement therapies [129,130]. Chronic GC administration, however, can lead to hypercortisolemia and associated adverse effects, including neuropsychiatric symptoms such as depression. These effects mirror those seen in endogenous hypercortisolism, such as that observed in Cushing syndrome [131] (156). Excessive or prolonged GC use also increases the risk of developing iatrogenic Cushing syndrome, further complicating the clinical picture. More than half of patients with Cushing syndrome experience depression, while up to three-quarters of those undergoing prolonged exogenous steroid therapy may exhibit mood alterations [123].

To mitigate these risks, GCs should be prescribed judiciously, ensuring their use is limited to well-established indications. Dosage should be carefully adjusted according to the patient’s age and weight, with attention to minimizing the duration and dose whenever possible [130]. Early recognition of depressive symptoms is crucial, as timely intervention can prevent progression and improve outcomes. Physicians should remain vigilant for the potential psychiatric side effects of GC therapy, including depression, to optimize patient safety and quality of life.

### 8.3. Osteopenia and Sarcopenia

One of the most prominent changes happening during aging is a loss in bone density and loss of muscle mass. Those processes are called osteopenia/osteoporosis and sarcopenia. Due to reduced bone and muscle mass, aging is associated with a higher risk of falls, fractures, and frailty. Osteoporosis is caused by an imbalance between bone reabsorption by osteoclasts and bone formation by osteoblasts. Different factors have an effect on bone formation and reabsorption, including sex, age, genetics, nutrients, physical activity, BMI, drugs, lifestyle, and chronic medical conditions [132].

Being the most common cause of secondary osteoporosis, long-term GC therapy negatively affects bone formation and reduces bone mass by suppressing the maturation, proliferation, and function of osteoblasts, stimulating osteoblast and osteocyte apoptosis, and extending osteoclast survival [133]. With the advancement of age, higher cortisol levels negatively affect bone formation and metabolism via the same mechanisms mentioned before [73,134]. Moreover, higher diurnal cortisol levels have been linked with frailty, a state of increased vulnerability. Conversely, lower diurnal cortisol levels are associated with longevity [17]. Postmenopausal women with higher salivary cortisol levels in the morning and the evening were more likely to suffer from sarcopenia, while those who had higher levels of DHEAS showed lower bone loss in a 15-year follow-up time [135,136]. Researchers have found that higher DHEA/S levels observed in the elderly are associated with increased bone density as well as greater muscle mass and strength, mobility, well-being, and physical functioning [17], while low DHEAS levels are associated with a higher prevalence of frailty and low back pain present in both sexes, a higher risk of non-vertebral fractures in aged men, and slower rehabilitation rates observed in women [14,137].

### 8.4. Insulin Resistance and Diabetes Mellitus

Aging is accompanied by impaired glucose tolerance and insulin resistance which can eventually lead to the occurrence of diabetes mellitus [138]. Moreover, quantitative and qualitative changes in insulin secretion in beta cells of pancreatic Langerhans islets significantly contribute to the pathogenesis of diabetes in aged patients [73]. Cortisol, as a catabolic hormone, raises blood sugar levels by stimulating gluconeogenesis in the liver and makes muscle and fat tissues less sensitive to insulin, resulting in higher blood sugar levels and insulin resistance [139]. Higher cortisol levels and flatter diurnal cortisol profiles in older people are associated with a higher risk of developing diabetes [95,140]. The effect of DHEA/S on insulin resistance is still inconsistent and more research needs to be conducted [14].

### 8.5. Immune Dysfunction

The immune system, like any other system, goes through extensive changes with increasing age and that process is called immunosenescence [76]. It involves inflammaging and the accumulation of senescent cells. Inflammaging represents the existence of chronic low-grade inflammation that advances with increasing age. Senescent cells acquire a specialized phenotype called secretory-associated senescent phenotype (SASP) which is responsible for the secretion of pro-inflammatory cytokines, growth factors, and chemokines. Those cells significantly contribute to the inflammaging leading to the development of age-related diseases [141,142]. It is well known that the elderly are more prone to infectious diseases and have a higher risk of severe outcomes. Moreover, advancing age is associated with an increased incidence of autoimmune diseases and tumors. All of this points out that immune function becomes dysfunctional with the advancing age which eventually leads to higher morbidity and mortality [143]. This happens due to complex changes affecting both innate and acquired immunity, including humoral and cellular components. Thymic involution, a decrease in naïve T and B cells, an increase in memory T and B cells, the dysfunctional activation of immune cells, and the predominance of pro-inflammatory cytokines are hallmarks of aging [142].

Cortisol and DHEA have opposite modulatory effects on the immune system and immune response. While cortisol is known to suppress the immune response, DHEA enhances it [17]. Cortisol has an anti-inflammatory and immunosuppressive effect. Higher cortisol levels are linked with boosting the function of suppressor T cells, lymphopenia, lower cytokine secretion, lymphoid organ atrophy, altered T cell traffic, and lower T cell proliferation [144]. On the other hand, DHEAS exerts an immunostimulatory effect, increasing the release of IL-2 by mitogen-stimulated CD4+ T cells [145]. Daily DHEA supplementation (50 mg) during the 20 weeks among the age-advanced males with low DHEAS levels showed a marked increase in IGF-I levels, immune cells (monocytes, B cells, ILC2, and NK cells), the mitogenic responses of both T and B cells, and the cytotoxicity of NK cells and cytokines. Moreover, patients who took DHEA supplementation showed a decreased CDR compared to the placebo group [146]. Higher CDR in the elderly is associated with a decline in immune functioning. Aged patients with hip fractures who had a higher CDR had increased infection rates, decreased neutrophil responses, and lower overall survival [17,75,147]. Therefore, it is proposed that the increase in cortisol and decrease in DHEA in the aged population may partly contribute to the increased susceptibility to infections, tumors, and inflammatory diseases.

## 9. Anti-Aging Strategies

Effective anti-aging strategies need to be introduced due to the ever-increasing number of people in the aging population. There are some drugs and therapeutic agents (resveratrol, rapamycin, and senolytics—dasatinib and quercetin) in the preclinical and clinical trials that show promising effects in modulating the aging process, but further research needs to be conducted before they can start being used for their anti-aging properties [148]. Even though many studies showed that DHEA/S have therapeutic and preventive effects on age-associated diseases, a 2-year randomized placebo control double-blind study with participants aged at least 60 years old failed to show any significant beneficial effect on body composition, physical performance, insulin sensitivity, or quality of life after a daily supplementation of 50 (for women) or 75 mg (for males) of DHEA. Also, DHEA replacement therapy has not shown any major adverse effects during the study [149]. Physical activity, calorie restriction, and stress management have been shown to positively affect the aging process and lower the CDR which results in better immune function, cognition, and well-being [148,150,151,152,153,154].

## 10. Conclusions

This review has explored the intricate interplay between cortisol, DHEA/S, obesity, pain perception, and aging, revealing several clinically significant insights. Hormonal imbalances, particularly elevated cortisol levels and reduced DHEA/S concentrations play a pivotal role in both acute and chronic pain while contributing to weight gain, accelerated aging, and age-related diseases. Dysregulation of the hypothalamic–pituitary–adrenal (HPA) axis emerges as a key factor in these processes, with consistent findings of altered cortisol awakening responses and disrupted diurnal rhythms.

Obesity is a complex condition involving genetic and hormonal factors. Dysregulated cortisol and DHEA secretion, along with the variations in hormone receptor expression and sensitivity, drive adipose tissue dysfunction, chronic inflammation, and metabolic disorders. Insulin resistance, dyslipidemia, and central adiposity heighten the risk of cardiovascular disease, musculoskeletal issues, and certain cancers. Continued research into these underlying mechanisms is essential to develop novel prevention and treatment strategies. Notably, weight reduction has been shown to significantly alleviate chronic pain and reduce morbidity, underscoring the urgent need for targeted therapies to address the multifaceted burden of obesity.

In pain management, the cortisol-to-DHEA ratio (CDR) shows potential as a biomarker for pain sensitivity, especially in surgical settings where higher ratios are associated with increased postoperative pain. This understanding may enable more individualized pain management strategies, including preoperative cortisol testing to identify patients at risk of heightened pain sensitivity and tailored interventions, such as psychotherapy or multimodal analgesia, to mitigate postoperative hyperalgesia. The mixed results regarding the correlation between postoperative pain and age underscore the need for further research in this area.

The role of DHEA/S as both a diagnostic indicator and therapeutic target is emphasized by its consistently reduced levels in chronic pain conditions and its novel mechanisms of action, such as modulation of Kv7 channels and neuronal activity. Gender-specific associations between DHEA/S levels and chronic pain further highlight its potential utility as a biomarker and therapeutic focus. Further empirical studies are essential to explore the potential of hormone replacement therapy targeting DHEA/S pathways as a novel approach to chronic pain management. While some evidence suggests that DHEA may influence pain modulation through various mechanisms, comprehensive clinical investigations are necessary to establish its efficacy and optimize treatment protocols. This focused research could significantly enhance our understanding of DHEA’s role in pain relief.

Aging is marked by rising cortisol levels and declining DHEA/S concentrations, contributing to age-related conditions such as cognitive decline, depression, bone density loss, metabolic disorders, and immune dysfunction. Elevated cortisol levels are particularly detrimental in neurodegenerative conditions, correlating with accelerated cognitive deterioration. These findings stress the importance of maintaining hormonal balance to support healthy aging, healthy weight, and effective pain management.

Standardizing hormonal assessment methodologies, such as blood, saliva, or urine testing, could provide reliable tools for both pain management and age-related disease prevention. Additionally, studies focusing on the relationship between CDR and acute pain, as well as the impact of preoperative mental health interventions on postoperative outcomes, could offer valuable insights.

By integrating hormonal assessments and interventions into clinical practice, this understanding of neuroendocrine dysfunction opens new opportunities for improving diagnostic accuracy, therapeutic strategies, and overall patient outcomes in the contexts of obesity, pain, and aging.

## Figures and Tables

**Table 1:** Changes in adrenal glands with increasing age.

Structural Changes	Functional Changes
Reduction in the zona reticularis	Increased cortisol levels
Concomitant increase in the zona fasciculata	Decreased DHEA and DHEAS levels
Discontinuous zona glomerulosa	Increased levels of 11-deoxy cortisol
Increased incidence of adrenal tumors	

**Table 2:** Changes in the HPA axis related to aging.

Age-Related HPA Axis Disruptions
Attenuated cortisol awakening response
Increased mean cortisol levels
Flattened diurnal cortisol secretion pattern
Higher evening and nighttime nadir
Shorter evening cortisol quiescent period
Decreased responsiveness to ACTH
Reduced steroid sulfation
Increased 11B-HSD1 activity in peripheral tissues
Decreased numbers of GRs
Lower CBG levels in plasma

## References

[1] One in Eight People Are Now Living with Obesity. https://www.who.int/news/item/01-03-2024-one-in-eight-people-are-now-living-with-obesity.

[2] Goldberg D.S., McGee S.J. (2011). Pain as a global public health priority. BMC Public Health.

[3] Sluka K.A., Wager T.D., Sutherland S.P., Labosky P.A., Balach T., Bayman E.O., Berardi G., Brummett C.M., Burns J., Buvanendran A. (2023). Predicting chronic postsurgical pain: Current evidence and a novel program to develop predictive biomarker signatures. Pain.

[4] Ageing and Health. https://www.who.int/news-room/fact-sheets/detail/ageing-and-health.

[5] Kamin H.S., Kertes D.A. (2017). Cortisol and DHEA in development and psychopathology. Horm. Behav..

[6] Mohd Azmi N.A.S., Juliana N., Azmani S., Mohd Effendy N., Abu I.F., Mohd Fahmi Teng N.I., Das S. (2021). Cortisol on Circadian Rhythm and Its Effect on Cardiovascular System. Int. J. Environ. Res. Public Health.

[7] Allen M.J., Sharma S. (2023). Physiology, Adrenocorticotropic Hormone (ACTH). StatPearls.

[8] Ehlert U., Gaab J., Heinrichs M. (2001). Psychoneuroendocrinological contributions to the etiology of depression, posttraumatic stress disorder, and stress-related bodily disorders: The role of the hypothalamus–pituitary–adrenal axis. Biol. Psychol..

[9] Thau L., Gandhi J., Sharma S. (2024). Physiology, Cortisol. StatPearls [Internet].

[10] Norman M., Hearing S.D. (2002). Glucocorticoid resistance—What is known?. Curr. Opin. Pharmacol..

[11] Sic A., Cvetkovic K., Manchanda E., Knezevic N.N. (2024). Neurobiological Implications of Chronic Stress and Metabolic Dysregulation in Inflammatory Bowel Diseases. Diseases.

[12] Nenezic N., Kostic S., Strac D.S., Grunauer M., Nenezic D., Radosavljevic M., Jancic J., Samardzic J. (2023). Dehydroepiandrosterone (DHEA): Pharmacological Effects and Potential Therapeutic Application. Mini Rev. Med. Chem..

[13] McKenna T.J., Fearon U., Clarke D., Cunningham S.K. (1997). A critical review of the origin and control of adrenal androgens. Baillieres Clin. Obstet. Gynaecol..

[14] Papadopoulou-Marketou N., Kassi E., Chrousos G.P., Feingold K.R., Anawalt B., Blackman M.R. (2000). Adrenal Androgens and Aging. Endotext [Internet].

[15] Clark B.J., Prough R.A., Klinge C.M. (2018). Mechanisms of Action of Dehydroepiandrosterone. Vitam. Horm..

[16] Tang J., Chen L.R., Chen K.H. (2021). The Utilization of Dehydroepiandrosterone as a Sexual Hormone Precursor in Premenopausal and Postmenopausal Women: An Overview. Pharmaceuticals.

[17] Yiallouris A., Tsioutis C., Agapidaki E., Zafeiri M., Agouridis A.P., Ntourakis D., Johnson E.O. (2019). Adrenal Aging and Its Implications on Stress Responsiveness in Humans. Front. Endocrinol..

[18] Ahmed T., Qassem M., Kyriacou P.A. (2023). Measuring stress: A review of the current cortisol and dehydroepiandrosterone (DHEA) measurement techniques and considerations for the future of mental health monitoring. Stress.

[19] Yen S.S., Laughlin G.A. (1998). Aging and the adrenal cortex. Exp. Gerontol..

[20] Ferrari E., Cravello L., Muzzoni B., Casarotti D., Paltro M., Solerte S.B., Fioravanti M., Cuzzoni G., Pontiggia B., Magri F. (2001). Age-related changes of the hypothalamic-pituitary-adrenal axis: Pathophysiological correlates. Eur. J. Endocrinol..

[21] Suh E., Cho A.R., Haam J.H., Gil M., Lee Y.K., Kim Y.S. (2023). Relationship between Serum Cortisol, Dehydroepiandrosterone Sulfate (DHEAS) Levels, and Natural Killer Cell Activity: A Cross-Sectional Study. J. Clin. Med..

[22] Morgan C.A., Southwick S., Hazlett G., Rasmusson A., Hoyt G., Zimolo Z., Charney D. (2004). Relationships Among Plasma Dehydroepiandrosterone Sulfate and Cortisol Levels, Symptoms of Dissociation, and Objective Performance in Humans Exposed to Acute Stress. Arch. Gen. Psychiatry.

[23] Bauer M.E. (2005). Stress, glucocorticoids and aging of the immune system. Stress.

[24] Lengton R., Schoenmakers M., Penninx B.W.J.H., Boon M.R., van Rossum E.F.C. (2024). Glucocorticoids and HPA axis regulation in the stress–obesity connection: A comprehensive overview of biological, physiological and behavioural dimensions. Clin. Obes..

[25] Lee M.J., Pramyothin P., Karastergiou K., Fried S.K. (2014). Deconstructing the roles of glucocorticoids in adipose tissue biology and the development of central obesity. Biochim. Biophys. Acta.

[26] Kumar R., Rizvi M.R., Saraswat S. (2022). Obesity and Stress: A Contingent Paralysis. Int. J. Prev. Med..

[27] Lengton R., Iyer A.M., van der Valk E.S., Hoogeveen E.K., Meijer O.C., van der Voorn B., van Rossum E.F.C. (2022). Variation in glucocorticoid sensitivity and the relation with obesity. Obes. Rev..

[28] Lee M.J., Fried S.K. (2014). The glucocorticoid receptor, not the mineralocorticoid receptor, plays the dominant role in adipogenesis and adipokine production in human adipocytes. Int. J. Obes..

[29] Vitellius G., Trabado S., Bouligand J., Delemer B., Lombès M. (2018). Pathophysiology of Glucocorticoid Signaling. Ann. Endocrinol..

[30] Majer-Łobodzińska A., Adamiec-Mroczek J. (2017). Glucocorticoid receptor polymorphism in obesity and glucose homeostasis. Adv. Clin. Exp. Med..

[31] Marzolla V., Armani A., Zennaro M.C., Cinti F., Mammi C., Fabbri A., Rosano G.M., Caprio M. (2012). The role of the mineralocorticoid receptor in adipocyte biology and fat metabolism. Mol. Cell Endocrinol..

[32] Infante M., Armani A., Marzolla V., Fabbri A., Caprio M. (2019). Adipocyte Mineralocorticoid Receptor. Vitam. Horm..

[33] Parasiliti-Caprino M., Bollati M., Merlo F.D., Ghigo E., Maccario M., Bo S. (2022). Adipose Tissue Dysfunction in Obesity: Role of Mineralocorticoid Receptor. Nutrients.

[34] Tchernof A., Labrie F. (2004). Dehydroepiandrosterone, obesity and cardiovascular disease risk: A review of human studies. Eur. J. Endocrinol..

[35] Karbowska J., Kochan Z. (2013). Effects of DHEA on metabolic and endocrine functions of adipose tissue. Horm. Mol. Biol. Clin. Investig..

[36] Ansari S., Haboubi H., Haboubi N. (2020). Adult obesity complications: Challenges and clinical impact. Ther. Adv. Endocrinol. Metab..

[37] Okifuji A., Hare B.D. (2015). The association between chronic pain and obesity. J. Pain. Res..

[38] Finnerup N.B., Kuner R., Jensen T.S. (2021). Neuropathic Pain: From Mechanisms to Treatment. Physiol. Rev..

[39] Lee G.I., Neumeister M.W. (2020). Pain: Pathways and Physiology. Clin. Plas Surg..

[40] Auyeung A., Wang H.C., Aravagiri K., Knezevic N.N. (2023). Kynurenine Pathway Metabolites as Potential Biomarkers in Chronic Pain. Pharmaceuticals.

[41] Li R., Chapman B.P., Smith S.M. (2021). Blood Dehydroepiandrosterone and Dehydroepiandrosterone Sulfate as Pathophysiological Correlates of Chronic Pain: Analyses Using a National Sample of Midlife Adults in the United States. Pain. Med..

[42] Yu H., Nagi S.S., Usoskin D., Hu Y., Kupari J., Bouchatta O., Yan H., Cranfill S.L., Gautam M., Su Y. (2024). Leveraging deep single-soma RNA sequencing to explore the neural basis of human somatosensation. Nat. Neurosci..

[43] Lerch J.K., Alexander J.K., Madalena K.M., Motti D., Quach T., Dhamija A., Zha A., Gensel J.C., Webster Marketon J., Lemmon V.P. (2017). Stress Increases Peripheral Axon Growth and Regeneration through Glucocorticoid Receptor-Dependent Transcriptional Programs. eNeuro.

[44] Tennant F. (2017). Cortisol Screening in Chronic Pain Patients. Pract. Pain. Manag..

[45] Opaleye T., Okoturo E., Adesina O.A., Oyapero A., Salami Y., Wemambu J.C. (2022). Salivary Cortisol as a Stress Monitor During Third Molar Surgery. J. Maxillofac. Oral. Surg..

[46] Fleszar M.G., Fortuna P., Zawadzki M., Hodurek P., Bednarz-Misa I., Witkiewicz W., Krzystek-Korpacka M. (2021). Sex, Type of Surgery, and Surgical Site Infections Are Associated with Perioperative Cortisol in Colorectal Cancer Patients. J. Clin. Med..

[47] Özmen Ö., Özçelik F., Kaygın M.A., Yılmaz H., Karakaya M.A. (2019). Evaluation of pain scoring and free cortisol levels of postoperative analgesic methods in cardiac surgery: A new perspective. Türk Göğüs Kalp Damar Cerrahisi Derg..

[48] Sun J., Xu W., Ye H., Tang D., Jiang Y., Kang Y., Pan J., Zhu J., Zhou M., Chen L. (2023). Stress Induces Prolonged Pain Recovery After Surgery: Involvement of Glucocorticoid-Related Pathway. Int. J. Neuropsychopharmacol..

[49] Benson S., Siebert C., Koenen L.R., Engler H., Kleine-Borgmann J., Bingel U., Icenhour A., Elsenbruch S. (2019). Cortisol affects pain sensitivity and pain-related emotional learning in experimental visceral but not somatic pain: A randomized controlled study in healthy men and women. Pain.

[50] Godfrey K.M., Herbert M., Strachan E., Mostoufi S., Crofford L.J., Buchwald D., Poeschla B., Succop A., Afari N. (2017). Dexamethasone-suppressed Salivary Cortisol and Pain Sensitivity in Female Twins. Clin. J. Pain.

[51] Knezevic E., Nenic K., Milanovic V., Knezevic N.N. (2023). The Role of Cortisol in Chronic Stress, Neurodegenerative Diseases, and Psychological Disorders. Cells.

[52] Telegan V.O., Tsagkaris C., Singh S.K., Tarasenko K.V. (2023). Subjective Assessments and Serum Cortisol Levels as Risk Factors of Pain Persistence in the Late Postoperative Period in Old and Oldest-Old Patients. Eur. J. Investig. Health Psychol. Educ..

[53] Cusack B., Buggy D.J. (2020). Anesthesia, analgesia, and the surgical stress response. BJA Educ..

[54] Ip H.Y.V., Abrishami A., Peng P.W.H., Wong J., Chung F. (2009). Predictors of Postoperative Pain and Analgesic Consumption. Anesthesiology.

[55] Khanna R., Slovacek H., Liles J., Haddad S., Poredos P., Bontekoe E., Jezovnik M., Hoppensteadt D., Fareed J., Hopkinson W. (2021). Regulation of Cortisol in Patients Undergoing Total Joint Arthroplasty. Clin. Appl. Thromb. Hemost..

[56] Lautenbacher S., Huber C., Kunz M., Parthum A., Weber P.G., Griessinger N., Sittl R. (2009). Hypervigilance as Predictor of Postoperative Acute Pain: Its Predictive Potency Compared With Experimental Pain Sensitivity, Cortisol Reactivity, and Affective State. Clin. J. Pain.

[57] Trevino C.M., Geier T., Morris R., Cronn S., deRoon-Cassini T. (2021). Relationship between Decreased Cortisol and Development of Chronic Pain in Traumatically Injured. J. Surg. Res..

[58] Yamamotova A., Kmoch V., Papezova H. (2012). Role of dehydroepiandrosterone and cortisol in nociceptive sensitivity to thermal pain in anorexia nervosa and healthy women. Neuro Endocrinol. Lett..

[59] Ahn R.S., Park J.W., Park I.S., Shin H.J., Ryu J.H., Oh A.Y., Park H.Y., Do S.H. (2022). The Involvement of the Hypothalamus-Pituitary-Adrenal Axis in the Development of Hyperalgesia during the Early Postoperative Period. Neuroendocrinology.

[60] Hannibal K.E., Bishop M.D. (2014). Chronic stress, cortisol dysfunction, and pain: A psychoneuroendocrine rationale for stress management in pain rehabilitation. Phys. Ther..

[61] Villafañe J.H., Pedersini P., Bertozzi L., Drago L., Fernandez-Carnero J., Bishop M.D., Berjano P. (2020). Exploring the relationship between chronic pain and cortisol levels in subjects with osteoarthritis: Results from a systematic review of the literature. Osteoarthr. Cartil..

[62] Generaal E., Vogelzangs N., Macfarlane G.J., Geenen R., Smit J.H., Penninx B.W., Dekker J. (2014). Reduced hypothalamic-pituitary-adrenal axis activity in chronic multi-site musculoskeletal pain: Partly masked by depressive and anxiety disorders. BMC Musculoskelet. Disord..

[63] Riva R., Mork P.J., Westgaard R.H., Lundberg U. (2012). Comparison of the cortisol awakening response in women with shoulder and neck pain and women with fibromyalgia. Psychoneuroendocrinology.

[64] Traish A.M., Kang H.P., Saad F., Guay A.T. (2011). Dehydroepiandrosterone (DHEA)—A Precursor Steroid or an Active Hormone in Human Physiology (CME). J. Sex. Med..

[65] Alhassen L., Alhassen W., Wong C., Sun Y., Xia Z., Civelli O., Hoshi N. (2023). Dehydroepiandrosterone Sulfate (DHEAS) Is an Endogenous Kv7 Channel Modulator That Reduces Kv7/M-Current Suppression and Inflammatory Pain. J. Neurosci..

[66] Semiz E.A., Hizmetli S., Semiz M., Karadağ A., Adalı M., Tuncay M.S., Alim B., Hayta E., Uslu A.U. (2016). Serum cortisol and dehydroepiandrosterone-sulfate levels after balneotherapy and physical therapy in patients with fibromyalgia. Saudi Med. J..

[67] Grimby-Ekman A., Ghafouri B., Sandén H., Larsson B., Gerdle B. (2017). Different DHEA-S Levels and Response Patterns in Individuals with Chronic Neck Pain, Compared with a Pain Free Group—A Pilot Study. Pain. Med..

[68] Yoon S., Roh D., Seo H., Kang S.Y., Han H.J., Beitz A.J., Lee J.H. (2009). Intrathecal injection of the neurosteroid, DHEAS, produces mechanical allodynia in mice: Involvement of spinal sigma-1 and GABA A receptors. Br. J. Pharmacol..

[69] Tennant F. (2013). The Physiologic Effects of Pain on the Endocrine System. Pain. Ther..

[70] López-Otín C., Blasco M.A., Partridge L., Serrano M., Kroemer G. (2013). The Hallmarks of Aging. Cell.

[71] Zhao J., Han Z., Ding L., Wang P., He X., Lin L. (2024). The molecular mechanism of aging and the role in neurodegenerative diseases. Heliyon.

[72] Pyo I.S., Yun S., Yoon Y.E., Choi J.W., Lee S.J. (2020). Mechanisms of Aging and the Preventive Effects of Resveratrol on Age-Related Diseases. Molecules.

[73] van den Beld A.W., Kaufman J.M., Zillikens M.C., Lamberts S.W.J., Egan J.M., van der Lely A.J. (2018). The physiology of endocrine systems with ageing. Lancet Diabetes Endocrinol..

[74] Gao X., Li F., Liu B., Wang Y., Wang Y., Zhou H. (2021). Cellular Senescence in Adrenocortical Biology and Its Disorders. Cells.

[75] Warde K.M., Smith L.J., Basham K.J. (2023). Age-related Changes in the Adrenal Cortex: Insights and Implications. J. Endocr. Soc..

[76] Stamou M.I., Colling C., Dichtel L.E. (2023). Adrenal aging and its effects on the stress response and immunosenescence. Maturitas.

[77] Hima L., Patel M.N., Kannan T., Gour S., Pratap U.P., Priyanka H.P., Vasantharekha R., ThyagaRajan S. (2020). Age-associated decline in neural, endocrine, and immune responses in men and women: Involvement of intracellular signaling pathways. J. Neuroimmunol..

[78] Liu P.Y., Reddy R.T. (2022). Sleep, testosterone and cortisol balance, and ageing men. Rev. Endocr. Metab. Disord..

[79] Deuschle M., Gotthardt U., Schweiger U., Weber B., Körner A., Schmider J., Standhardt H., Lammers C.H., Heuser I. (1997). With aging in humans the activity of the hypothalamus-pituitary-adrenal system increases and its diurnal amplitude flattens. Life Sci..

[80] Boscaro M., Paoletta A., Scarpa E., Barzon L., Fusaro P., Fallo F., Sonino N. (1998). Age-Related Changes in Glucocorticoid Fast Feedback Inhibition of Adrenocorticotropin in Man. J. Clin. Endocrinol. Metab..

[81] Carroll B.J., Feinberg M., Greden J.F., Tarika J., Albala A.A., Haskett R.F., James N.M., Kronfol Z., Lohr N., Steiner M. (1981). A specific laboratory test for the diagnosis of melancholia. Standardization, validation, and clinical utility. Arch. Gen. Psychiatry.

[82] Bini J., Bhatt S., Hillmer A.T., Gallezot J.D., Nabulsi N., Pracitto R., Labaree D., Kapinos M., Ropchan J., Matuskey D. (2020). Body Mass Index and Age Effects on Brain 11β-Hydroxysteroid Dehydrogenase Type 1: A Positron Emission Tomography Study. Mol. Imaging Biol..

[83] Mueller J.W., Gilligan L.C., Idkowiak J., Arlt W., Foster P.A. (2015). The Regulation of Steroid Action by Sulfation and Desulfation. Endocr. Rev..

[84] Tezuka Y., Atsumi N., Blinder A.R., Rege J., Giordano T.J., Rainey W.E., Turcu A.F. (2021). The Age-Dependent Changes of the Human Adrenal Cortical Zones Are Not Congruent. J. Clin. Endocrinol. Metab..

[85] Pavlov E.P., Harman S.M., Chrousos G.P., Loriaux D.L., Blackman M.R. (1986). Responses of Plasma Adrenocorticotropin, Cortisol, and Dehydroepiandrosterone to Ovine Corticotropin- Releasing Hormone in Healthy Aging Men. J. Clin. Endocrinol. Metab..

[86] Davio A., Woolcock H., Nanba A.T., Rege J., O’Day P., Ren J., Zhao L., Ebina H., Auchus R., Rainey W.E. (2020). Sex Differences in 11-Oxygenated Androgen Patterns Across Adulthood. J. Clin. Endocrinol. Metab..

[87] Nanba A.T., Rege J., Ren J., Auchus R.J., Rainey W.E., Turcu A.F. (2019). 11-Oxygenated C19 Steroids Do Not Decline With Age in Women. J. Clin. Endocrinol. Metab..

[88] Heaney J.L.J., Phillips A.C., Carroll D. (2012). Ageing, physical function, and the diurnal rhythms of cortisol and dehydroepiandrosterone. Psychoneuroendocrinology.

[89] Enomoto M., Adachi H., Fukami A., Furuki K., Satoh A., Otsuka M., Kumagae S., Nanjo Y., Shigetoh Y., Imaizumi T. (2008). Serum dehydroepiandrosterone sulfate levels predict longevity in men: 27-year follow-up study in a community-based cohort (Tanushimaru study). J. Am. Geriatr. Soc..

[90] Aldred S., Mecocci P. (2010). Decreased dehydroepiandrosterone (DHEA) and dehydroepiandrosterone sulfate (DHEAS) concentrations in plasma of Alzheimer’s disease (AD) patients. Arch. Gerontol. Geriatr..

[91] Villareal D.T., Holloszy J.O. (2006). DHEA enhances effects of weight training on muscle mass and strength in elderly women and men. Am. J. Physiol-Endocrinol. Metab..

[92] Gorelik S.G., Belousova O.N., Treneva E.V., Bulgakova S.V., Zakharova N.O., Nesterenko S.A. (2022). Effect of Daily Rhythms of Cortisol Secretion on the Rate of Aging in Men. Arch. Razi Inst..

[93] Chatzi G., Chandola T., Shlomo N., Cernat A., Hannemann T. (2024). Socioeconomic position and HPA axis activity among older adults living in England. Psychoneuroendocrinology.

[94] Zilioli S., Jiang Y., Byrd D., Joseph N. (2023). Lifetime discrimination, habitual and daily everyday discrimination, and diurnal cortisol among older African Americans adults. Psychoneuroendocrinology.

[95] Gaffey A.E., Bergeman C.S., Clark L.A., Wirth M.M. (2016). Aging and the HPA axis: Stress and resilience in older adults. Neurosci. Biobehav. Rev..

[96] Demakakos P., Steptoe A. (2022). Adverse childhood experiences and diurnal cortisol patterns in older people in England. Psychoneuroendocrinology.

[97] Behfar Q., Ramirez Zuniga A., Martino-Adami P.V. (2022). Aging, Senescence, and Dementia. J. Prev. Alzheimer Dis..

[98] Scheltens P., De Strooper B., Kivipelto M., Holstege H., Chételat G., Teunissen C.E., Cummings J., van der Flier W.M. (2021). Alzheimer’s disease. Lancet.

[99] Lyons C.E., Bartolomucci A. (2020). Stress and Alzheimer’s disease: A senescence link?. Neurosci. Biobehav. Rev..

[100] Lavretsky H., Newhouse P.A. (2012). Stress, Inflammation, and Aging. Am. J. Geriatr. Psychiatry.

[101] Piazza J.R., Almeida D.M., Dmitrieva N.O., Klein L.C. (2010). Frontiers in the use of biomarkers of health in research on stress and aging. J. Gerontol. B Psychol. Sci. Soc. Sci..

[102] Wirth M., Lange C., Huijbers W. (2019). Plasma cortisol is associated with cerebral hypometabolism across the Alzheimer’s disease spectrum. Neurobiol. Aging.

[103] Dronse J., Ohndorf A., Richter N., Bischof G.N., Fassbender R., Behfar Q., Gramespacher H., Dillen K., Jacobs H.I.L., Kukolja J. (2023). Serum cortisol is negatively related to hippocampal volume, brain structure, and memory performance in healthy aging and Alzheimer’s disease. Front. Aging Neurosci..

[104] Vyas S., Rodrigues A.J., Silva J.M., Tronche F., Almeida O.F., Sousa N., Sotiropoulos I. (2016). Chronic Stress and Glucocorticoids: From Neuronal Plasticity to Neurodegeneration. Neural Plast..

[105] Lupien S.J., Fiocco A., Wan N., Maheu F., Lord C., Schramek T., Tu M.T. (2005). Stress hormones and human memory function across the lifespan. Psychoneuroendocrinology.

[106] Ouanes S., Clark C., Richiardi J., Maréchal B., Lewczuk P., Kornhuber J., Kirschbaum C., Popp J. (2022). Cerebrospinal Fluid Cortisol and Dehydroepiandrosterone Sulfate, Alzheimer’s Disease Pathology, and Cognitive Decline. Front. Aging Neurosci..

[107] Eachus H., Ryu S. (2024). Glucocorticoid effects on the brain: From adaptive developmental plasticity to allostatic overload. J. Exp. Biol..

[108] Feeney J., Newman L., Kenny R.A. (2021). Hair glucocorticoids and resting-state frontal lobe oxygenation: Findings from The Irish Longitudinal Study on Ageing. Psychoneuroendocrinology.

[109] Arbo B.D., Ribeiro F.S., Ribeiro M.F. (2018). Astrocyte Neuroprotection and Dehydroepiandrosterone. Vitam. Horm..

[110] Gardner M., Lightman S., Kuh D., Comijs H., Deeg D., Gallacher J., Geoffroy M.C., Kivimaki M., Kumari M., Power C. (2019). Dysregulation of the hypothalamic pituitary adrenal (HPA) axis and cognitive capability at older ages: Individual participant meta-analysis of five cohorts. Sci. Rep..

[111] Martocchia A., Gallucci M., Noale M., Maggi S., Cassol M., Stefanelli M., Postacchini D., Proietti A., Barbagallo M., Dominguez L.J. (2022). The increased cortisol levels with preserved rhythmicity in aging and its relationship with dementia and metabolic syndrome. Aging Clin. Exp. Res..

[112] Ennis G.E., An Y., Resnick S.M., Ferrucci L., O’Brien R.J., Moffat S.D. (2017). Long-term cortisol measures predict Alzheimer disease risk. Neurology.

[113] Zheng B., Tal R., Yang Z., Middleton L., Udeh-Momoh C. (2020). Cortisol hypersecretion and the risk of Alzheimer’s disease: A systematic review and meta-analysis. Ageing Res. Rev..

[114] Peña-Bautista C., Baquero M., Ferrer I., Hervás D., Vento M., García-Blanco A., Cháfer-Pericás C. (2019). Neuropsychological assessment and cortisol levels in biofluids from early Alzheimer’s disease patients. Exp. Gerontol..

[115] de Leeuw M., Verhoeve S.I., van der Wee N.J.A., van Hemert A.M., Vreugdenhil E., Coomans C.P. (2023). The role of the circadian system in the etiology of depression. Neurosci. Biobehav. Rev..

[116] Alexopoulos G.S. (2005). Depression in the elderly. Lancet.

[117] Meyers B.S., Alexopoulos G.S. (1988). Geriatric depression. Med. Clin. N. Am..

[118] Adam E.K., Quinn M.E., Tavernier R., McQuillan M.T., Dahlke K.A., Gilbert K.E. (2017). Diurnal cortisol slopes and mental and physical health outcomes: A systematic review and meta-analysis. Psychoneuroendocrinology.

[119] Ho R.T.H., Fong T.C.T., Yau J.C.Y., Chan W.C., Kwan J.S.K., Chiu P.K.C., Lam L.C.W. (2020). Diurnal Cortisol Slope Mediates the Association Between Affect and Memory Retrieval in Older Adults With Mild Cognitive Impairment: A Path-Analytical Study. Front. Aging Neurosci..

[120] Deuschle M., Weber B., Colla M., Depner M., Heuser I. (1998). Effects of major depression, aging and gender upon calculated diurnal free plasma cortisol concentrations: A re-evaluation study. Stress..

[121] Lupien S.J., Nair N.P., Brière S., Maheu F., Tu M.T., Lemay M., McEwen B.S., Meaney M.J. (1999). Increased cortisol levels and impaired cognition in human aging: Implication for depression and dementia in later life. Rev. Neurosci..

[122] Sahu P., Gidwani B., Dhongade H.J. (2020). Pharmacological Activities of Dehydroepiandrosterone: A Review. Steroids.

[123] Herbert J. (1998). Neurosteroids, brain damage, and mental illness. Exp. Gerontol..

[124] Belvederi Murri M., Pariante C., Mondelli V., Masotti M., Atti A.R., Mellacqua Z., Antonioli M., Ghio L., Menchetti M., Zanetidou S. (2014). HPA axis and aging in depression Systematic review and meta-analysis. Psychoneuroendocrinology.

[125] Gupta D., Morley J.E. (2014). Hypothalamic-pituitary-adrenal (HPA) axis and aging. Compr. Physiol..

[126] Ferrari E., Casarotti D., Muzzoni B., Albertelli N., Cravello L., Fioravanti M., Solerte S.B., Magri F. (2001). Age-related changes of the adrenal secretory pattern possible role in pathological brain aging. Brain Res. Brain Res. Rev..

[127] Sekhon S., Patel J., Sapra A. (2023). Late-Life Depression. StatPearls [Internet].

[128] Brown E.S., Chandler P.A. (2020). Mood and cognitive changes during systemic corticosteroid therapy. Prim Care Companion J. Clin. Psychiatry.

[129] Hodgens A., Sharman T. (2023). Corticosteroids. StatPearls [Internet].

[130] Schäcke H., Döcke W.D., Asadullah K. (2002). Mechanisms involved in the side effects of glucocorticoids. Pharmacol. Ther..

[131] Reincke M., Fleseriu M. (2023). Cushing Syndrome: A Review. JAMA.

[132] Aibar-Almazán A., Voltes-Martínez A., Castellote-Caballero Y., Afanador-Restrepo D.F., Carcelén-Fraile M.D.C., López-Ruiz E. (2022). Current Status of the Diagnosis and Management of Osteoporosis. Int. J. Mol. Sci..

[133] Weinstein R.S. (2012). Glucocorticoid-Induced Osteoporosis and Osteonecrosis. Endocrinol. Metab. Clin. N. Am..

[134] Hofbauer L.C., Rauner M. (2009). Minireview: Live and Let Die: Molecular Effects of Glucocorticoids on Bone Cells. Mol. Endocrinol..

[135] Gonzalez Rodriguez E., Marques-Vidal P., Aubry-Rozier B., Papadakis G., Preisig M., Kuehner C., Vollenweider P., Waeber G., Hans D., Lamy O. (2021). Diurnal Salivary Cortisol in Sarcopenic Postmenopausal Women: The OsteoLaus Cohort. Calcif. Tissue Int..

[136] Ghebre M.A., Hart D.J., Hakim A.J., Kato B.S., Thompson V., Arden N.K., Spector T.D., Zhai G. (2011). Association between DHEAS and bone loss in postmenopausal women: A 15-year longitudinal population-based study. Calcif. Tissue Int..

[137] Ohlsson C., Nethander M., Kindmark A., Ljunggren Ö., Lorentzon M., Rosengren B.E., Karlsson M.K., Mellström D., Vandenput L. (2017). Low Serum DHEAS Predicts Increased Fracture Risk in Older Men: The MrOS Sweden Study. J. Bone Miner. Res..

[138] Jones C.M., Boelaert K. (2014). The Endocrinology of Ageing: A Mini-Review. Gerontology.

[139] Ward A.M., Fall C.H., Stein C.E., Kumaran K., Veena S.R., Wood P.J., Syddall H.E., Phillips D.I. (2003). Cortisol and the metabolic syndrome in South Asians. Clin. Endocrinol..

[140] Schoorlemmer R.M., Peeters G.M., van Schoor N.M., Lips P. (2009). Relationships between cortisol level, mortality and chronic diseases in older persons. Clin. Endocrinol..

[141] Quiros-Roldan E., Sottini A., Natali P.G., Imberti L. (2024). The Impact of Immune System Aging on Infectious Diseases. Microorganisms.

[142] Liu Z., Liang Q., Ren Y., Guo C., Ge X., Wang L., Cheng Q., Luo P., Zhang Y., Han X. (2023). Immunosenescence: Molecular mechanisms and diseases. Signal Transduct. Target. Ther..

[143] Müller L., Di Benedetto S., Pawelec G. (2019). The Immune System and Its Dysregulation with Aging. Subcell. Biochem..

[144] Vitlic A., Lord J.M., Phillips A.C. (2014). Stress, ageing and their influence on functional, cellular and molecular aspects of the immune system. Age.

[145] Dillon J.S. (2005). Dehydroepiandrosterone, dehydroepiandrosterone sulfate and related steroids: Their role in inflammatory, allergic and immunological disorders. Curr. Drug Targets Inflamm. Allergy.

[146] Khorram O., Vu L., Yen S.S.C. (1997). Activation of Immune Function byDehydroepiandrosterone (DHEA) in Age-Advanced Men. J. Gerontol. A Biol. Sci. Med. Sci..

[147] Butcher S.K., Killampalli V., Lascelles D., Wang K., Alpar E.K., Lord J.M. (2005). Raised cortisol:DHEAS ratios in the elderly after injury: Potential impact upon neutrophil function and immunity. Aging Cell.

[148] Li Z., Zhang Z., Ren Y., Wang Y., Fang J., Yue H., Ma S., Guan F. (2021). Aging and age-related diseases: From mechanisms to therapeutic strategies. Biogerontology.

[149] Nair K.S., Rizza R.A., O’Brien P., Dhatariya K., Short K.R., Nehra A., Vittone J.L., Klee G.G., Basu A., Basu R. (2006). DHEA in elderly women and DHEA or testosterone in elderly men. N. Engl. J. Med..

[150] Elraiyah T., Sonbol M.B., Wang Z., Khairalseed T., Asi N., Undavalli C., Nabhan M., Altayar O., Prokop L., Montori V.M. (2014). The Benefits and Harms of Systemic Dehydroepiandrosterone (DHEA) in Postmenopausal Women With Normal Adrenal Function: A Systematic Review and Meta-analysis. J. Clin. Endocrinol. Metab..

[151] De Nys L., Ofosu E.F., Ryde G.C., Connelly J., Whittaker A.C. (2023). Physical Activity Influences Cortisol and Dehydroepiandrosterone (Sulfate) Levels in Older Adults: A Systematic Review and Meta-Analysis. J. Aging Phys. Act..

[152] Zouhal H., Jayavel A., Parasuraman K., Hayes L.D., Tourny C., Rhibi F., Laher I., Abderrahman A.B., Hackney A.C. (2021). Effects of Exercise Training on Anabolic and Catabolic Hormones with Advanced Age: A Systematic Review. Sports Med..

[153] Morucci G., Ryskalin L., Pratesi S., Branca J.J.V., Modesti A., Modesti P.A., Gulisano M., Gesi M. (2022). Effects of a 24-Week Exercise Program on Functional Fitness, Oxidative Stress, and Salivary Cortisol Levels in Elderly Subjects. Medicina.

[154] Traustadóttir T., Bosch P.R., Matt K.S. (2005). The HPA Axis Response to Stress in Women: Effects of Aging and Fitness. Psychoneuroendocrinology.

